# Clinical performance of the LMA Protector™ airway in moderately obese patients

**DOI:** 10.1186/s12871-020-01100-z

**Published:** 2020-07-31

**Authors:** Ina Ismiarti Shariffuddin, Sook Hui Chaw, Ling Wei Ng, Ching Hooi Lim, Mohd Fitry Zainal Abidin, Wan A. Wan Zakaria, Wendy H. Teoh

**Affiliations:** 1 Department of Anesthesiology, Faculty of Medicine, University of Malaya, 50603 Kuala Lumpur, Malaysia; 2 Wendy Teoh Pte.Ltd, Private Anaesthesia Practice, Singapore, Singapore

**Keywords:** Supraglottic airway devices, Moderately obese, Oropharyngeal leak pressures, Airway management

## Abstract

**Background:**

The 4th National Audit Project of The Royal College of Anaesthetists and The Difficult Airway Society (NAP4) reported a higher incidence of supraglottic airway device (SAD) related pulmonary aspiration in obese patients especially with the first-generation SADs. The latest single-use SAD, the Protector™ provides a functional separation of the respiratory and digestive tracts and its laryngeal cuff with two ports allowing additional suction in tandem with the insertion of a gastric tube. The laryngeal cuff of LMA Protector™ allows a large catchment reservoir in the event of gastric content aspiration.

**Methods:**

We evaluated the performance characteristics of the LMA Protector™ in 30 unparalysed, moderately obese patients. First attempt insertion rate, time for insertion, oropharyngeal leak pressure (OLP), and incidence of complications were recorded.

**Results:**

We found high first and second attempt insertion rates of 28(93%) and 1(33%) respectively, with one failed attempt where no capnography trace could be detected, presumably from a downfolded device tip. The LMA Protector™ was inserted rapidly in 21.0(4.0) seconds and demonstrated high OLP of 31.8(5.4) cmH2O. Fibreoptic assessment showed a clear view of vocal cords in 93%. The incidence of blood staining on removal of device was 48%, postoperative sore throat 27%, dysphagia 10% and dysphonia 20% (all self-limiting, resolving a few hours postoperatively).

**Conclusions:**

We conclude that the LMA Protector™ was associated with easy, expedient first attempt insertion success, demonstrating high oropharyngeal pressures and good anatomical position in the moderately obese population, with relatively low postoperative airway morbidity.

**Trial registration:**

Australian New Zealand Clinical Trials Registry, ACTRN12617001152314. Registered 7 August 2017.

## Background

Obesity is a known risk factor associated with many complications in anaesthesia, including difficult airway. The Fourth National Audit Project of the Royal College of Anaesthetist and Difficult Airway Society (NAP4) has reported a higher incidence of supraglottic airway device (SAD) associated pulmonary aspiration in obese patients, especially with the first-generation SAD [1].

The LMA Protector™ is the latest single-use second-generation SAD made from medical-grade silicone. It has a preformed-fixed curved structure for easy insertion. It provides access and functional separation of the respiratory and digestive tracts. The respiratory channel can be used as a direct intubation conduit. Uniquely, the LMA Protector contains two drain channels, which emerge as separate ports proximally. A suction tube may be attached to the male drainage port around the laryngeal region or a well-lubricated gastric tube may be passed through the female drainage port to the stomach. The high leakage pressures, the optimal fit of the airway device, and dual gastric ports offer better protection from aspiration [2]. Besides, these features support high ventilation pressures and this would be ideal for obese patients.

Currently, there are limited reports on the outcome of the LMA Protector in airway management and its use in the obese patients. Hence, we conducted this study to evaluate the performance of the LMA Protector in obese (BMI 30–35) patients who required general anaesthesia for surgical procedures.

## Methods

Approval from the University of Malaya Medical Centre’s Institutional Review Board (201755–5215) and the Australian New Zealand Clinical Trials Registry (ACTRN12617001152314) were obtained before the conduct of the study.

We recruited moderately obese (30 kg/m^2^ ≤ BMI ≤ 35 kg/m^2^) patients who were scheduled for elective open surgical procedures under general anaesthesia amenable to supraglottic airway device insertion. Exlusion criteria were patients with American Society of Anaesthesiologists (ASA) physical status IV, those at high risk of aspiration (symptomatic gastro-esophageal reflux and hiatus hernia), recent upper respiratory tract infection, previous head and neck surgery or radiotherapy and small mouth opening.

The patients were not premedicated preoperatively. They were positioned supine on the operating table, with the head resting on a head ring. Standard monitoring were applied before induction of anaesthesia. The insertion of LMA Protector was done based on manufacturer’s recommendation. The LMA Protector cuff was completely deflated and a water-based lubricant was applied to the posterior part of cuff and airway tube. Only the LMA protector size 3 or 4 was selected for use in our patients, size 3 for woman and size 4 for man.

After pre-oxygenation, anaesthesia was induced with fentanyl 1.5–2 mcg/kg, propofol 2–3 mg/kg, and anaesthesia were maintained with sevoflurane (end tidal concentration of 2 to 3%) in 100% oxygen until minimum alveolar concentration (MAC) of 1.2 and patient’s jaw was considered relaxed at the discretion of the investigators, before insertion of the LMA. Under direct vision, the tip of the device was pressed flat against the hard palate and the LMA Protector was inserted until resistance was felt. The cuff was then inflated with air until the marker of the pilot balloon was within the green zone (indicative of 40-60 cm H_2_0, with an upper limit of clear zone where the pressure does not exceed 70cmH_2_0). The amount of air inflated was recorded, and the intra-cuff pressure was confirmed with a handheld aneroid manometer (Portex® Pressure Gauge; Smiths Medical Intl Ltd., Kent, UK) to achieve an intra-cuff pressure of 40- 60cmH_2_O.

The time of insertion was measured from when the tip of the LMA entered the patient’s mouth to the time of appearance of first square end tidal carbon dioxide (ETCO^2^), denoting successful establishment of effective ventilation. Otherwise, the device was removed for another insertion attempt. Each attempt was defined as re-insertion of the airway device into the mouth. A maximum of three insertion attempts were allowed. “Insertion failure” occurred when the investigators failed 3 attempts of insertion or if the entire process of insertion exceeded 120 s. In case of insertion failure, the attending anaesthesiologists would decide the subsequent airway management.

Once the airway device was in place, the SAD was fixed by taping over the patient’s cheek. A gel plug was placed in the male gastric drain outlet whilst closing the female port of the gastric drain and the suprasternal notch test was done to confirm placement (gently tapping the suprasternal notch causes the gel to pulsate, confirming the tip location behind the cricoid cartilage). Then, a pre-lubricated 14 French gauge gastric tube was inserted through the female port gastric drain, and graded 1 to 3 (1-easy, 2-difficult, 3-impossible). Confirmation of correct placement of the gastric tube was done by auscultating the epigastrium as air was injected, and by aspiration of gastric contents. We decompressed the stomach and the amount of gastric fluid aspirated was documented, and the fasting duration recorded.

The anatomical airway position of the LMA Protector was then assessed by fibreoptic bronchoscopy (3.7 mm bronchoscope, Karl Storz™, Tuttlingen, Germany) via the airway channel and scored as follows: grade 4, only vocal cords seen; grade 3, vocal cords and posterior epiglottis seen; grade 2, vocal cords and anterior epiglottis seen; and grade 1, vocal cords not seen [3].

Oropharyngeal leak pressure (OLP) was measured after closing the adjustable pressure-limiting (APL) valve with a fresh gas flow of 3 L min^− 1^, noting the airway pressure at equilibrium or when there was an audible air leak from the throat. The epigastrium was also auscultated when measuring the OLP to detect any air entrainment in the stomach. The blood pressure and heart rate were also recorded every minute for the first 5 minutes from beginning of insertion of the LMA Protector™^.^ Airway manoeuvres to facilitate the insertion of the LMA were also documented.

Anaesthesia was maintained with an oxygen and air mixture in sevoflurane to achieve MAC of 1.0–1.2. Patients were placed in supine or lithotomy position based on the types of procedures. All intraoperative complications, such as desaturation to less than 95%, regurgitation or aspiration, bronchospasm, dental, lip or tongue injury, were recorded. At the end of surgery, the airway device was removed upon the adequate spontaneous breathing and eye opening of the patient. The airway device was inspected for the presence of blood. Forty-five minutes later, an independent observer assessed the patients for post-operative sore throat, dysphonia, and dysphagia.

All SAD insertions were performed by anaesthesiologists with more than 10-years’ experience in supraglottic airway management and had performed at least ten LMA Protector™ insertion before the study commencement (IIS, CSH and MFZA) [4]. Data collection was done by another independent investigator.

### Statistical analysis

Our primary measure was “first attempt success rate”. Sample size was based on assumption that 95% of the subjects in the population have first attempt success rate insertion of LMA Protector. Hence, the study would require a sample size of 19 with 10% margin of error relative to the expected proportion and 95% confidence [5]. Therefore, we recruited 30 patients to account for dropouts and protocol breaches. The data that was collected was analysed using SPSS 24 (SPSS Inc., Chicago, IL, USA). Mean and standard deviation was used to describe normally distributed continuous variables; median and interquartile ranges (IQR) were used for non-normally distributed continuous variables. Categorical variables were expressed as number and percentage.

## Results

Thirty patients were recruited from August 2017 to August 2018. The performance of LMA protector was evaluated. The patients’ baseline demographics, airway anthropometric features, and duration of surgery is depicted in Table 1.

**Table 1:** Baseline demographic and airway anthropometric features

Parameters (*n* = 30)	Results
Age, years	43.3 (16.7)
Body Mass Index, kg/m2	31.7 (1.4)
Gender	
Male	10 [33.3%]
Female	20 [66.7%]
ASA status	
1	15 [50%]
2	15 [50%]
Types of surgery (Open surgeries)	
General surgery	10 [33.3%]
Orthopaedic surgery	9 [30.0%]
Gynaecology	6 [20.0%]
Urology	5 [16.7%]
Mallampati	
1	12 [40.0%]
2	14 [46.7%]
3	4 [13.3%]
4	0 [0%]
Thyromental distance	
> 60 mm	29 [96.7%]
< 60 mm	1 [3.3%]
Interincisor distance	
> 40 mm	30 [100%]
< 40 mm	0 [0%]
Neck flexion	
Full	29 [96.7%]
< 50% limited	0 [0%]
> 50% limited	1 [3.3%]
Neck extension	
Full	29 [96.7%]
< 50% limited	0 [0%]
> 50% limited	1 [3.3%]
Duration of surgery, minutes	71.8 (39.8)

Data expressed as mean (standard deviation) or number [percentage]

*ASA* American Society of Anesthesiologists

**Fig. 1:**
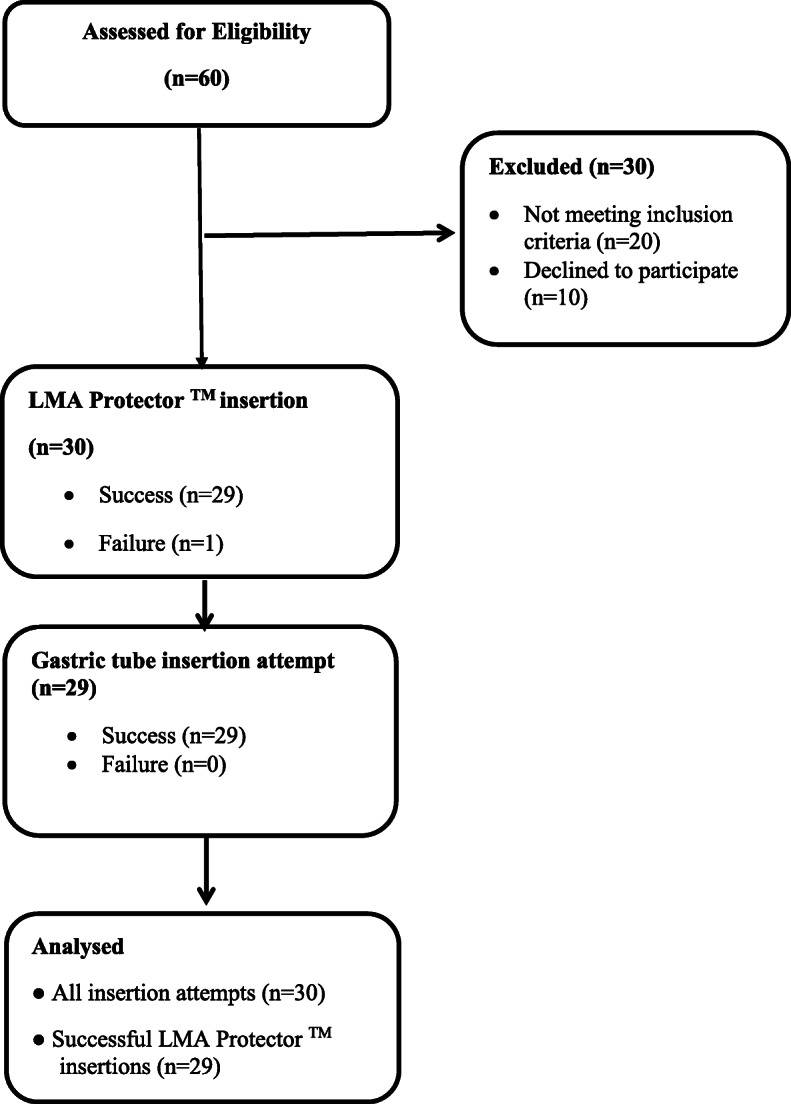
Flow chart for patients’ recruitment and data analysis

Insertion of the LMA Protector™ was successful in 28 patients (93%) at first attempt and one patient (3%) at second attempt. However, there was an insertion failure in one patient, despite application of rescue manoeuvres. This patient was subsequently managed with another SGA for ventilation. We excluded this patient from the analysis. (Fig. 1).

The mean time taken for insertion was 21.0 (4.0) seconds. For all the successful LMA insertions, the ease of LMA Protector™ insertion was graded as easy in 26 patients and fair in 3 patients.

The LMA protector™ was found to provide a good seal with mean OLP of 31.8 (5.4) mmHg. Insertion of a gastric tube was easy in 23 patients (79%), while 6 (21%) patients had a difficult gastric tube insertion. On examination of fibreoptic view, 27 patients had a clear view of their vocal cords (93%), 1 had view of arytenoids only, and 1 had view of epiglottis only (Table 2).

**Table 2:** Parameters for the clinical performance of LMA Protector™

Parameters (n = 30)	Results
Size of LMA protector	
Size 3	10 [63.3%]
Size 4	11 [36.7%]
Number of attempts	
1	28 [93.4%]
2	1 [3.3%]
Failed	1 [3.3%]
Ease of insertion	
Easy	26 [86.7%]
Fair	3 [10.0%]
Difficult	0
Very Difficult	0
Impossible	1 [3.3%]
Time to successful insertion, seconds	21.0 (3.9)
Oropharyngeal leak pressure, OLP, cmH20	31.8 (5.4)
Cuff volume, mls	14.6 (5.6)
Ease of gastric tube insertion	
Easy	23 [79.3%]
Difficult	6 [20.7%]
Impossible	0
Volume of gsastric fluid aspirated, ml	6.9 (13.1)
Duration of fasting, hours	11.7(2.6)
Fibreoptic view	
Only vocal cords seen	27 [93.1%]
Vocal cords and posterior epiglottis	1 [3.4%]
Vocal cords and anterior epiglottis	1 [3.4%]
Vocal cord not seen	0

Data expressed as mean (standard deviation) or number [percentage]

There were no airway complications related to SAD insertion during maintenance of anaesthesia. (Table 3) Mucosal injury as evidenced by blood stains on the LMA Protector™ was documented in 48% of patients. Delayed complications were post-operative sore throat (28%), post-operative dysphagia (21%), and post-operative dysphonia (10%). The degree of post-operative sore throat, dysphagia and dysphonia were described to be mild in nature and lasted for less than 24 h. Most patients did not have any increase in heart rate and mean arterial pressure of more than 20% when compared to baseline, except in 2 patients (7%) (Table 4). There was no incidence of desaturation in all patients.

**Table 3:** Complications related to LMA Protector insertions

Complications	Percentage
Immediate	
Desaturation	0
Bronchospasm	0
Gross regurgitation/aspiration	0
Intraoperative gastric distension	0
Dental injury	0
Lip or tongue injury	0
Mucosal injury/blood on LMA	14 [48.4%]
Delayed	
Post operative sore throat	8 [27.6%]
Post operative dysphonia	3 [10.3%]
Post operative dysphagia	6 [20.7%]

Data expressed as mean (standard deviation) or number [percentage]

**Table 4:** Patients’ haemodynamic response to LMA Protector™ airway insertion

Parameters (n = 30)	Results
Heart Rate	
Increase of 0–10%	18 [62.1%]
Increase of 10–20%	9 [31.0%]
Increase of > 20%	2 [6.9%]
Mean arterial pressure	
Increase of 0–10%	23 [79.3%]
Increase of 10–20%	2 [6.9%]
Increase of > 20%	2 [6.9%]

Data expressed as mean (standard deviation) or number [percentage]

## Discussion

In our study, we found a high first attempt success rate (93%) of LMA Protector™ insertion in thirty moderately obese patients which was comparable to previous studies using LMA Protector™ in non-obese patients [6, 7]. Use of second-generation SADs with a gastric channel and design that allow fibreoptic guided tracheal intubation is crucial in obese patients as it provides efficient airway protection from gastric aspiration, as well as a conduit for intubation in case of an unexpected difficult airway [8, 9].

The average BMI in our study was 32. All female patients received the size 3 LMA Protector, and males the size 4. Their ideal mean (SD) body weights of males 66.9 (4.4) kg and females 46.5 (7.3) kg suggested a nice fit according to manufacturer recommendations that we followed e.g., size 3 for less than 50 kg and size 4 for patients weighing in between 50 to 70 kg^.2^. Previous studies using Proseal LMA™ that had utilised the size 4 for females and size 5 for males, found that despite yielding higher oropharyngeal leak pressures, the larger mask tended to rise up within the mouth more often, predisposing these patients to increased risk of sore throat and lingual nerve damage [10].

The insertion of the LMA Protector™ was graded easy/fair for 97% of our patients. The LMA Protector™ was inserted expediently in a mean time of 21.0 (4.0) seconds which is comparable to studies using LMA Supreme™ and Ambu® AuraGain™ [11]. The comparable device insertion times shows that there is no increased difficulty in insertion in obese patients compared to non-obese patients despite its bulkier profile with the 2 gastric ports. This is reassuring, as the LMA Protector™ is a new airway device and investigators had only 10 insertions before commencing the study; it is possible that increased usage and experience with the LMA Protector™ could further improve the success rate and insertion timings [4, 6, 12].

We had one failed insertion where despite an easy insertion, we could not obtain a capnograph tracing after 3 attempts. For the first and second insertion attempt, the investigator (CSH) had used the index finger to guide insertion of the LMA Protector™ which was totally deflated and generously lubricated according to manufacturer’s recommendations. The entry of the device was smooth but there was absence of end tidal carbon dioxide trace and chest rise upon commencing ventilation. Prior to the third insertion attempt, the patient’s head and neck was repositioned, laryngospasm was ruled out, and adequate depth of anaesthesia was confirmed, but again failed to yield an end tidal carbon dioxide trace. Insertion failure was declared, and the airway was successfully rescued with an Ambu® AuraGain™. Being made from medical grade silicone that renders the LMA Protector™ softer and more pliable in nature compared to the polyvinyl chloride (PVC) tip of the Ambu AuraGain, we postulated that the tip of the LMA Protector™ had folded over in the posterior pharynx during insertion. In hindsight, a diagonal shift of the mask during insertion may have been helpful to avoid this downfolding [2].

We found a high OLP of 31.8 (5.4) cmH_2_0 in this study, which is higher than that reported in non-obese patients. Moser et al. reported an OLP of 28.3 (7.0) cmH_2_0 while Sng et al. reported an OLP of 25.5 cmH_2_0 (IQR 23.0 to 29.0 cmH_2_0) [6, 7]. This high OLP is similar to the OLP of LMA Proseal which was reported to be 27 (7.0) cmH_2_0 but higher than that of Ambu AuraGain 24.1 (7.4) cmH_2_0 [11, 13]. The high OLP can be attributed to the fact that the LMA Protector™ is made of medical grade silicone, with an anatomically shaped airway tube and inflatable cuff that purportedly conforms to the contours of an individual’s hypopharynx. In obese patients, the increased adiposity and hypopharyngeal tissue may render it a snugger fit. Obese patients have poor chest compliance due to their thick chest wall, and often require higher peak inspiratory pressures when positive pressure ventilation is instituted [1]. This high OLP of the LMA Protector™ therefore makes it a suitable SGA to be used in obese patients as it is beneficial to mitigate any air leak that may predispose patients to inadequate ventilation, gastric insufflation, and increased risk of aspiration. The insertion of gastric drain tube into the oesophagus was rated easy in 79% and with the tip of the gastric channel aligned with the oesophagus, there was effective venting of gastric content.

On assessment of the anatomical position of the LMA Protector™, we found a clear view of the vocal cords in 93% of the patients. This could be attributed to the anatomically curved tube of the LMA Protector™ enabling insertion to the optimal position. A good anatomical position, in conjunction with high sealing pressures enables obese patients to be ventilated more safely even with higher peak pressures. A good anatomical position also makes it an effective conduit for endotracheal tube insertion in these obese patients, either as a rescue procedure after difficult or failed initial laryngoscopy, or in those individuals who require a conversion from a SAD to tracheal tube for their surgeries or further postoperative ventilation in the ICU.

In two patients, we did not have an entirely clear view of the vocal cords. The vocal cords were partially seen with the posterior epiglottis in one patient, and partial VC with anterior epiglottis sighted in the other patient. No problems with ventilation were encountered and during maintenance of anaesthesia over a mean surgical duration of 71 min, the LMA Protector™ performed well without the need for airway manipulation to optimize ventilation.

Van Zundert et al. similarly found a high OLP with the LMA protector of 31.7 (2.9) cm H2O as we did. Uniquely, their device insertions were performed under vision of a video laryngoscope using an ‘insert-detect-correct-as-you-go’ technique with standardized corrective measures, and they achieved a near-optimal fibreoptic position in the LMA-Protector of 94%, similar to our results [14]. This is reassuring as our study showed that simple manual insertion of the LMA Protector in obese patients, without “vision” adjuncts, worked just as well.

We had assessed immediate and delayed post-operative complications associated with the LMA Protector™ in our study. The only complication encountered during the immediate phase was mucosal injury, seen in 14 patients (48%). The incidence was high compared to figures from non-obese patients [7]. The airway of an obese patient is shown on MRI to have deposition of excess adipose tissue into nearly all pharyngeal structures including the uvula, the tonsils, the tonsillar pillars, the tongue, aryepiglottic folds, and most predominantly, the lateral pharyngeal walls. This leads to airway narrowing, which can be exaggerated by external compression from superficial depositions of fat in the neck [15]. Therefore, at insertion of LMA, the pharyngeal structures could be easily abraded especially if there is concomitant tissue congestion. Another possible reason for the increased incidence of mucosal injury could be the larger tip of the LMA Protector that may collide with the arytenoids upon its insertion. Additionally, the bulky posterior curvature of LMA Protector and its slightly larger cuff may contribute to a more challenging insertion in the Asian population with their smaller builts and mouth opening in this study. Ensuring a well lubricated LMA Protector and a completely deflated cuff before insertion is paramount.

The LMA Protector™ pilot balloon has an Integrated Cuff Pilot™ which is used for intraoperative cuff pressure monitoring. The provision of continuous intra-cuff pressure monitoring is ideal as intracuff pressure could change, at a given volume, because of temperature changes, muscular tone or administration of nitrous oxide [16]. The use of the Integrated Cuff Pilot™ can prevent nerve or pressure injuries of the airway especially with prolonged usage. We followed up our patients via phone call after discharge from hospital and found a 27% incidence of postoperative sore throat, which was comparable to a study using Proseal LMA™ in obese patients [17]. It was self-limiting and only lasted for a few hours postoperatively. We found a lower incidence of dysphagia (10%) and dysphonia (20%) compared to Rieger et al. [18] This can perhaps be attributed to the continuous cuff pressure monitoring with the integrated cuff pressure indicator, which is targeted between 40 and 60 mmHg. In addition, the cuff is made of medical grade silicone, which increases its flexibility and hence potentially more pliable and less traumatic to insert into the pharynx [6]. We also did not find any hypoglossal nerve injury with the use of LMA Protector™ as reported by Tham et al. [19]

Most patients did not have a significant increase in heart rate and mean arterial pressure when compared to baseline, except two patients (7%). This is consistent with a previous study reporting stable haemodynamics as LMA insertion is easy and less stimulating to the patients [19].

Our study had a few limitations. Firstly, we evaluated the LMA Protector™ in obese patients with BMI of 30–35, and the results cannot be extrapolated to patients beyond BMI > 35. Secondly, our study sample size was a relatively small cohort number, albeit powered adequately. Thirdly, surgical duration in our study lasted a mean of 71 min, with the longest duration 180 min. Any incidence of dysphonia beyond that is still unknown. But our results are reassuring in support of the LMA Protector’s use in the obese.

## Conclusion

This evaluation study showed that the LMA Protector™ was associated with easy, expedient first attempt insertion success, demonstrating high oropharyngeal pressures and good anatomical position in the moderately obese population, with relatively low postoperative airway morbidity with good spontaneous recovery.

## Data Availability

The datasets used and/or analyzed during the current study are available from the corresponding author on reasonable request.

## Abbreviations

LMA: Laryngeal mask airway
SAD: Supraglottic airway device
OLP: Oropharyngeal leak pressure
ASA: American Society of Anaesthesiologist
ETCO^2^: End tidal carbon dioxide
APL: Adjustable pressure-limiting
IQR: Interquartile range
PVC: Polyvinyl chloride
